# On the Role of Chinese English as a Foreign Language: Teachers’ Well-Being and Loving Pedagogy in Their Academic Engagement

**DOI:** 10.3389/fpsyg.2022.941226

**Published:** 2022-07-08

**Authors:** Bo Li, Guoxin Miao

**Affiliations:** ^1^Public Foreign Language Teaching and Research Department, Jilin University of Finance and Economics, Changchun, China; ^2^College of the Humanities, Jilin University, Changchun, China

**Keywords:** academic engagement, Chinese EFL teachers, teachers’ well-being, loving pedagogy, English as a foreign language

## Abstract

Positive emotions are regarded as vital issues in English as a foreign language (EFL) instruction. This study attempted to consider the relationships between Chinese EFL teachers’ psychological well-being, loving Pedagogy, and work engagement as the constructs of positive psychology in academic contexts. It also tried to examine the contribution of psychological well-being and loving pedagogy in work engagement. To this end, 414 Chinese EFL teachers including participated in this study. The three questionnaires called Dispositions toward Loving Pedagogy Scale, Index of Psychological Well-Being at Work, and Self-report Engagement Questionnaire were distributed among learners. The findings showed significant relationships between well-being, loving pedagogy, and work engagement. Moreover, the results indicated that teachers’ psychological well-being significantly predicted their work engagement. This study provided some implications for teachers, teacher educators, and educational policy-makers to raise their awareness of adopting loving pedagogy and boosting teacher well-being for the enhancement of teacher involvement in academic contexts.

## Introduction

As with field of educational psychology, positive psychology as a new branch emerged and focused on personal growth. Positive psychology is a reaction against psycho-analysis and behaviorism, which have focused on mental illness, meanwhile emphasizing maladaptive behavior and negative thinking (Li, 2020). Positive psychology is “the scientific study of what makes life most worth living” (Peterson et al., 2008) or “the scientific study of positive human functioning and flourishing on multiple levels that include the biological, personal, relation an institutional, cultural, and global dimensions of life” (Seligman and Csikszentmihalyi, 2000). Positive psychology builds further on the humanistic movement, which encouraged an emphasis on happiness, well-being, and positivity, thus creating the foundation for what is now known as positive psychology. This new branch of psychology includes components such as positive teacher–student relationship, self-efficacy, well-being, enjoyment, grit, and engagement (Wang et al., 2021).

In order to succeed and survive in a continuously changing and competitive environment, schools need motivated proactive and initiative teachers who collaborate. In fact, schools that try to engage teachers who have an affective bond with their professional activities surpass others. The concept of work engagement was first coined by Kahn (1990), who defined it as the “harnessing of organization members’ selves to their work roles. In engagement, people employ and express themselves physically, cognitively, emotionally, and mentally during role performances” (p. 694). In fact, it is the extent to which an individual is attentive and absorbed in the performance of his or her work. According to Kahn (1990), when people are engaged, they are not only physically involved in their work, but also are cognitively alert and emotionally connected to others at the moment of engagement. Educators with higher levels of engagement in their job are inclined to be active, devoted, and fascinated by the educational contexts (Seligman, 2011). Work engagement, as a positive psychological construct, has drawn the attention of man investigators in recent years (Buric and Macuka, 2018). Greenier et al. (2021) stated that teachers’ psychological well-being is considered as the main reason for the involvement of educators in academic contexts. Keyes et al. (2002) defined psychological well-being as an “individual’s awareness that he or she has, or will have, a meaningful and self-fulfilling life” (p. 5).

As an educational and emotional activity, teaching comprised of teachers’ teaching and students’ learning through which completing the interaction between the instructor and the learner is required (Li et al., 2019). Consequently, in order for teachers to get their own teaching responsibilities fulfilled, they need to be in contact with their students, that is, the establishment of a relationship between teacher and students is mandatory (Pöysä et al., 2018). The nature and quality of teachers’ relationships with their students play a crucial and central role in motivating and engaging students to learn (Martin and Collie, 2019). Teacher–student interpersonal relationships are fundamental to students’ academic, social and emotional development, and accordingly may affect the social and learning environments of classrooms and schools (Li et al., 2019). A teacher–student relationship is described as “the emotional bond student and teacher share with each other” (Newberry and Davis, 2008, p. 1966), in which the quality of the relationship is controlled by how strong the bond is. The characteristics of both student and teacher can form and change the quality of relationships (Sabol and Pianta, 2012). Wubbels et al. (2014) defined teacher–student relationship as “the generalized interpersonal meaning students and teachers attach to their interactions with each other” (Wubbels et al., 2014, p. 364). Teacher–student relationship is defined by Brinkworth et al. (2018) as teachers’ and students’ ongoing perceptions of one another, which are stored in memory and guide future actions and interactions. Teachers’ and students’ individual personalities and classroom milieu are constructs which may affect teacher–student relationship but they are different from teacher–student relationship (Brinkworth et al., 2018). Indeed, instruction and education are affected by emotional states (Sikma, 2021). Love, as another significant positive emotional construct which is related to teacher–student relationship, has been scarcely investigated in instructive contexts. Määttä and Uusiautti (2011) emphasized love as a significant aspect of education. They considered pedagogical love as one of the facets of human love. Seligman (2002) described pedagogical love as the teachers’ attention to learners’ needs. Yin et al. (2019) also defined pedagogical love as care, sensitivity, and empathy among teachers and students regarding their needs, experiences, and development.

Concepts such as psychological well-being, pedagogical love, and work engagement were considered to be significant variables in improving the performances of the teachers in the literature. However, there have been few previous investigations of teachers’ work engagement which make it necessary for investigators to do research in this field. Having an awareness of teachers’ well-being and pedagogical love, and their roles in work engagement can endorse and expand the positive psychology constructs.

Moreover, the achievement or incompetence in improving language learning depends on some particular vital variables, including social and cultural environment, nature, and instructional method, and contextual features (Torre et al., 2020). Numerous investigations were done on teacher–learner interpersonal relationship along with notable efforts to understand its effect on teachers and learners. The significance of this study is to raise educators’ awareness of their emotional behaviors and their impacts on their work engagement. Moreover, by knowing about teachers’ pedagogical love and well-being, school managers would be able to make their teachers enthusiastic and improve their level of engagement. The objective of this study is to investigate the effect of Chinese English as a foreign language (EFL) teachers’ well-being and loving pedagogy on their academic engagement. To this end, this study tries to answer the following questions:

1. Are there any significant relationships between Chinese EFL teachers’ well-being, loving pedagogy, and their work engagement?
2. Do Chinese EFL teachers’ well-being and loving pedagogy significantly predict their work engagement?

## Literature Review

### Work Engagement

Numerous definitions have been provided for work engagement which considers various dimensions (Greenier et al., 2021). According to Bakker et al. (2008), work engagement refers to “a positive, fulfilling, affective-motivational state of work-related well-being” (p. 187). However, Shuck and Wollard (2010) defined work engagement as “an individual’s cognitive, emotional, and behavioral state directed toward desired organizational outcomes” (p. 103). According to these definitions, work engagement can support individuals to involve rigorously in their work and probably lessen or decrease job burnout. This is the point that Maslach and Leiter (1997) asserted in their conceptualization of working engagement as a construct that is located at the end of the burnout-engagement continuum based on individuals’ satisfaction and work involvement. In another definition, Smith et al. (2012) regarded work engagement as an emotional factor in one’s emotional states, and it is regarded considered to be one’s approach to his/her occupation, which influences the performance. Moreover, May et al. (2004) conceptualized work engagement as an individual’s optimistic, satisfying, and job-related emotional states described by vigor, dedication, and absorption. They stated that vigor denotes the higher levels of energy, the inclination to accomplish in a job, and resilience in coping with adversaries. They maintained that dedication is related to the individual’s intense engagement in one’s career, and experiencing a sense of significance, enthusiasm, inspiration, pride, and challenge. Lastly, absorption occurs when an individual draws his/her attention to a job with full concentration and confidence. In an educational context, Fredricks (2013) defined engagement as the degree of learners’ and educators’ commitment and investment in their performance. He also maintained that engagement is regarded as an umbrella term that consists of learners’ and educators’ degree of devotion, concentration, and inclination to use abilities, approaches, or activities to develop their performance.

Investigating teacher engagement is crucial since involved teachers are efficient in instruction, and they can boost learner engagement (Bakker and Bal, 2010). Louis and Smith (1992) pointed out that “in primary or secondary education, teacher engagement refers to a teacher’s psychological investment in an effort toward teaching the knowledge, skills, and crafts he or she wishes students to master” (p. 120). Raina and Khatri (2015) stated that some factors, such as educational experience, learners’ aptitude, class size, school location, class the school, classroom contexts, classroom management, task management, novelties in educational contexts and instruction, feedback received by learners and principal, interaction with colleagues, and opportunities for cooperation with others are critical in teacher engagement. Timms and Brough (2013) emphasized the importance of two theoretical models for explaining teachers’ levels of work engagement, namely, Demerouti et al.’s (2001) job-demands-resources model and Ryan and Deci’s (2001) self-determination model. They asserted that the job-demands-resources model, compared to the self-determination one, is widely used in educational contexts since it is applied to explain teacher burnout along with job involvement.

Traditionally, psychologists were concerned primarily with the students’ and educators’ negative emotional states, and they tried to diminish them (Derakhshan et al., 2021). In recent years, positive psychology, as a modern approach in educational contexts, attempted to enlighten the favorable educational circumstances and contexts for the accomplishment of students and educators (Jiang, 2020). Therefore, the attention shifted from burnout to work engagement to improve its possible outcomes in the workplace (Buric and Macuka, 2018). Earlier investigations have mainly focused on the negative emotions of language teachers in numerous cultural contexts, because of the challenging nature of instruction (King and Ng, 2018). However, some studies have found a significant correlation between working engagement and other positive psychological constructs, such as enjoyment and hope (Ghanizadeh and Moafian, 2010), self-efficacy (Song et al., 2018), and resiliency (Perera et al., 2018). In order to highlight the significant effect of positive constructs of psychology in foreign language instruction, and to emphasize the inspection of educators’ positive emotions and experiences, this investigation ponders into the predictive role of psychological well-being and loving pedagogy, as two positive psychological constructs, in language educators’ work engagement.

### Psychological Well-Being

Pathology-oriented perspectives, which highlight the effect of negative emotions on individuals’ performance, have been criticized by the positive psychologists who attempted to increase well-being by focusing on the strengths of individuals (Seligman and Csikszentmihalyi, 2000). MacIntyre et al. (2019a) stated that positive psychologist tries to enhance human positive emotions, which results in success, well-being, and improvement in their actions and practices. MacIntyre et al. (2019b) stated that well-being is considered one of the main reasons that individuals are involved in instruction or education. Barry et al. (2017) defined well-being as “a process of acquiring a set of skills to recognize and manage emotions, set and achieve positive goals, appreciate others’ perspectives, establish and maintain positive relationships, make responsible decisions, and handle interpersonal situations constructively” (p. 21). Keyes (2002) categorized individuals’ well-being in two approaches, known as subjective well-being and psychological well-being measured by hedonic and eudemonic methods, respectively. Diener et al. (2010) pointed out that subjective well-being concerns with the development of enjoyment and avoiding agony. On the other hand, Carr (2013) defined psychological well-being as one’s potential to have a meaningful and fulfilling life. Ryff (1995), in another definition, stated that well-being is a way of enjoyment and it is defined as “striving for perfection that represents the realization of one’s true potential” (p. 100). Garg and Rastogi (2009) also stated that psychological well-being is related to an individual’s enjoyment of his/her mental and physical health, lifetime, job, and judgment of one’s contentment in life. Ryff (1989) proposed a model for psychological well-being including numerous components like autonomy, environmental domination, personal growth, positive relationship, purposes in life, and accepting the self. They stated that autonomy is related to freedom, self-regulation, self-determination, and concentration on one’s internal control. Environmental domination is defined as developing appropriate context based on individualized and spiritual situations. He also defined personal growth as individuals’ capability to constantly develop their behaviors and sense of identity, and advance their aptitudes during their lifetime. Moreover, he included positive relationships as an aspect of psychological well-being since it expands a trusting, loving, and sincere rapport with other individuals. Purposes in life are also defined as the individuals’ belief to have objectives, meanings, and targets for their life. Finally, he asserted that self-acceptance refers to the individuals’ acceptance of their qualities, and having a non-judgmental consideration of the positive and negative dimensions of the personality throughout a lifetime.

Few investigations in the past have been done to inspect the relationship between psychological well-being and work-related variables such as work engagement. Zeng et al. (2019), in their study in the Chinese context, demonstrated that teachers’ growth mindset, well-being, and resilience strongly predict job engagement. They used Fredrickson’s (2001) broaden-and-build theory of positive emotions to pinpoint that “the experience of positive emotions served to broaden the scope of people’s attention, thought processes, and action; and in long-term, build physical, intellectual and social resources” (p. 7). They argued that cheerful individuals tend to use opportunities at work, and they are inclined to discover and adapt to new situations, which raise their vigor and absorption at work. They also found out that well-being and grit mediate the correlation between work engagement and growth mindset. Shuck and Reio (2014) found that higher work engagement among individuals increased their psychological well-being and individual achievement. They argued that when individuals feel the positivity of their work environment, they draw from individual-level outcome resources, and involve more in their job. Sudibjo and Sutarji (2020), in their study, revealed that teachers’ psychological well-being, job satisfaction, and emotional intelligence significantly predict their work engagement. Greenier et al. (2021) examined British and Iranian English teachers’ emotion regulation and psychological well-being and their relationship with work engagement. They found that British and Iranian English teachers’ emotion regulation and psychological well-being significantly predicted work engagement. Using broad-and-build theory, they justified this correlation by arguing that teacher satisfaction can extend persons’ thought-action ranges and develop their capabilities, thus increasing their psychological well-being and ideal performance. In other words, they mentioned that engaged teachers tend to experience enjoyment and satisfaction in their job.

Teacher’s wellbeing is the leading cause for the development of teaching quality, learners’ academic achievement, building positive relationships with language learners along with their own job satisfaction (MacIntyre et al., 2019b). Therefore it is vital to appreciate the nature of teacher well-being, as a relatively new construct, and the factors which correlate with it (King and Ng, 2018). This study tries to shed light on upcoming investigations on the issue in educational fields.

### Pedagogical Love

Many definitions have been provided for the word “love.” Berscheid (2006) pointed out that “the word love is used in an astounding array of situations to describe an enormous range of attitudes, emotions, feelings, and behavior toward objects and people” (p. 172). Määttä and Uusiautti (2011) categorized different types of love into maternal and paternal love, romantic love, love for fellow humans, love of one’s country, and pedagogical love. Seligman (2011) defined love, as a construct proposed by positive psychologists, which is assumed to be a fundamental human need that promotes individuals’ academic performance. In similar vein, Wang et al. (2021) indicated that one of the constructs of positive psychology is the pedagogical love, which provides a pleasant learning experience for educators and learners. Zhao and Li (2021) defined pedagogical love, as a well-known construct, in language instruction that concerns instructors’ compassion, friendliness, empathy, and care toward their learners’ emotions, requirements, education, and accomplishment. According to their definition, educators’ and learners’ inner feelings and the specific contexts where they perform their tasks are not detached from each other. Pavelescu and Petrić (2018) asserted that a loving educator has the capability to boost learners’ language learning and motivation. Moreover, Dowling (2014) defined pedagogical love as a natural and robust desire which influences learners’ affective dimensions and interactions.

Grimmer (2021) stated that taking on a pedagogical love builds up students’ self-sufficiency, activity, confidence, critical thinking, and positive relationship with teachers and peers. Yin et al. (2019) asserted that teaching is not limited to the transfer of knowledge and should be beyond academic outcomes. They stated that effective teaching occurs in a loving environment to increase learners’ emotional status, classroom interaction, motivation, social ability, personality, and mental health. They also believed that education and instruction stem from pedagogical love. They maintained that educators can employ loving pedagogy in their instruction to instruct learners through love. In this respect, Loreman (2011) proposed a theoretical model of pedagogical love by considering the religious, emotional, and logical dimensions of human life. He incorporated some positive features, including kindness, passion, empathy, bonding, intimacy, sacrifice, forgiveness, and community, and acceptance into pedagogical love. He mentioned that love can bring out positive educational experiences. Barcelos and Coelho (2016) also stated that pedagogy of love is an integration of some positive psychological constructs, such as ethics, growth, care, respect, freedom, and dialog. Moreover, they maintained that approving learners’ emotional states and aptitudes and considering language and love as two critical components of language learners’ academic development will contribute educators considering the efficiency of love in their methodologies and instructional techniques.

Barcelos (2020) stated that practicing pedagogical love can have some positive results for EFL learners and educators including their enhanced motivation, activity, self-sufficiency, social communication skills, creativity, resilience, academic performance, academic engagement, pedagogical success, etc. Barcelos and Coelho (2016) also indicated the limitation of research on the use of pedagogical love in language education since it is a new topic for second language acquisition (SLA) practitioners. In the field of SLA, Wang et al. (2022) highlighted the conceptualization and application of loving pedagogy in SLA research and practice. They proposed a practical model of loving pedagogy for SLA practitioners. Page (2018) also suggested a tentative model for practitioners to implement pedagogical love in SLA in educational contexts.

Engels et al. (2021), in their study, revealed that intimacy, as a component of pedagogical love, and conflict, significantly affected learners’ academic engagement. They mentioned that learners’ feelings about the support of teachers can inspire them to engage socially, academically, and emotionally in educational contexts. Derakhshan (2021) indicated that empathic instructors play ethical roles for their learners through helping them engage in interactions with their peers. He argued that such interaction fosters the educational quality and helps learners enhance their positive behaviors. Hashim et al. (2014) verified the significant relationship between teacher–learner rapport and learners’ engagement. They argued that English teachers’ love and empathy toward learners and their well-being can increase learners’ tendency to participate in educational contexts. Their study also implicated that instructors should emphasize pleasant qualities including truthfulness, admiration, empathy, and sensitivity to learners’ requirements, since these ideals fundamentally generate learning motivation and engagement. Bullough (2019) also stated that teacher empathy not only increases learner engagement, but also contributes teachers to attaining social justice across different contexts. Mercer and Dörnyei (2020) asserted that learner engagement is instructor-dependent and it is a dynamic construct. They argued that instructors can take important actions to enhance EFL learners’ commitment. Consequently, they can show empathy, compassion, kindness, and numerous kinds of support to improve learners’ behavioral and emotional engagement. Khan and Armstrong (2019) argued that the enhancement of compassion, empathy, and sympathy among teachers are the critical issues that should be considered to develop learner engagement and create pleasant performance among learners. These studies support the significant effect of pedagogical love on learners’ engagement. However, investigating the engagement on the part of teachers and the role of adopting pedagogical love in shaping this construct can raise educators’ awareness in the educational context.

## Methodology

### Participants

The participants of this study were 414 teachers including both genders (male = 121/29.02%; female = 296/70.98%) with different academic qualifications undergraduate = 184/44.12%, postgraduate = 120/28.78%, doctor = 26/6.24% and years of teaching experiences with 1–5, 6–10, 11–15, 16–20, and 21 years plus accounting for 120/28.78%, 81/19.42%, 48/11.51%, 80/19.18%, and 88/21.1%, respectively. With an average of 42, their age ranged from 17 to 87. They were selected from different colleges, institutes, and universities in China, with the majority in Jilin Province (316/75.77%), one of the provinces of northeast China and the other 21 provinces (98/24.23%) *via* Wenjuanxing (an online data-collection program). Giving consent was informed to all participants. All responses were based on their own willingness.

### Data Collection Procedures

By online distribution of three questionnaires of Loving Pedagogy Scale, Index of Psychological Well-Being at Work, and Self-report Engagement Questionnaire, data was smoothly collected in the middle of February. Altogether, 417 questionnaires (414 valid) were gleaned from different colleges, universities and institutes in various provinces of China. Before participating in this study, all teachers had been acquainted with filling in the questionnaire *via* WeChat or other technical devices and had been informed of their rights and freedom of withdrawal from this study if they sensed any discomfort. The guarantee was made that all collected data would only be used for research purposes. All participants had made no contact with any researcher, so there would not be any human-intervened data or interest conflict. To increase the reliability and validity of the study, the questionnaires were presented in English which were translated and examined for any potential misunderstanding by three experts in linguistics, translation, and psychology. Then, the collected data was cleaned before being sent into SPSS for further analysis, which paved the way for the probe into the research question.

## Results

In order to decide on the data analysis, preliminary measurements should be done. The first step is to measure the reliability of the three questionnaires used in this study.

To measure the reliability indices of all three questionnaires, Table 1 indicates that the process of calculation was repeated three times and the outputs of Cronbach’s alpha revealed that the Psychological Well-being questionnaire (*r* = 0.97), Loving Pedagogy questionnaire (*r* = 0.98), and Work Engagement questionnaire (*r* = 0.97) had satisfactory reliability indices.

**TABLE 1:** Reliability of the questionnaires.

Questionnaire	Cronbach’s alpha	N of items
Psychological well-being	0.97	25
Loving pedagogy	0.98	29
Work engagement	0.97	17

One of the ways the researcher used for making a decision about using parametric or non-parametric analysis in a quantitative study, is to measure the normality of the data. Table 2 shows the Kolmogorov–Smirnov index which shows that the distribution of data is not normal for any of the variables (sig = 0.000). The assumption for having a normal set of data is to have a non-significant index of K–S, but the output revealed that the data normality rule is violated for psychological well-being, loving pedagogy, work engagement, and a non-parametric analysis should be conducted to calculate the possible relationships among the variables.

**TABLE 2:** Test of normality for psychological well-being, loving pedagogy, work engagement.

	Kolmogorov–Smirnov^a^			Shapiro–Wilk		
	Statistic	df	Sig.	Statistic	df	Sig.
PWB	0.174	417	0.000	0.900	417	0.000
LP	0.227	417	0.000	0.840	417	0.000
WE	0.095	417	0.000	0.947	417	0.000

*^a^Lilliefors significance correction.*

### The First Research Question

The first research question was posed to measure the possible relationship between Chinese EFL teachers’ psychological well-being, loving pedagogy, work engagement. Since it was revealed in the previous Table that the data are not normal, a non-parametric correlation index (Spearman Rho) was used.

Based on the correlational rules, the greater the amount of the relationship, the stronger the possibility of a significant relationship. As shown in Table 3, the relationship between Chinese EFL teachers’ psychological well-being and work engagement, is direct and significant (*r* = 0.607, *P* = 0.000). Similarly, the relationship between Teachers’ loving pedagogy, and work engagement is direct and significant (*r* = 0.700, *P* = 0.000). It can be concluded that the higher the level of teachers’ loving pedagogy, and psychological well-being, the higher their level of work engagement.

**TABLE 3:** Correlation among for psychological well-being, loving pedagogy, work engagement.

Correlations					
Spearman’s rho	PWB		PWB	LP	WE
		Correlation coefficient	1.000	0.700**	0.607**
		Sig. (two-tailed)		0.000	0.000
	LP	N	417	417	417
		Correlation coefficient	0.700**	1.000	0.530**
		Sig. (two-tailed)	0.000		0.000
	WE	N	417	417	417
		Correlation coefficient	0.607**	0.530**	1.000
		Sig. (two-tailed)	0.000	0.000	

**Correlation is significant at the 0.01 level (two-tailed).

### The Second Research Question

The second research question concerns the extent to which Chinese EFL teachers’ psychological well-being and loving pedagogy can predict their work engagement. This measurement was done though running a multiple regression analysis. The following tables were the output of linear multiple regression analysis including, model summary, ANOVA, and coefficient.

Table 4 provides a model summary for Chinese EFL teachers’ psychological well-being, loving pedagogy, and work engagement. It was shown that the model, which contains the scores of teachers’ PWB and LP can explain the amount of variance in the dependent variable (teachers’ work engagement). This model can explain 42.2% of the variances in the teachers’ work engagement.

**TABLE 4:** Model summary for psychological well-being, loving pedagogy, work engagement.

Model	*R*	*R* square	Adjusted *R* square	Std. error of the estimate
1	0.650	0.422	0.419	13.33

*^a^Predictors: (Constant), PWB, LP.*

Table 5 labeled ANOVA tested the hypothesis that multiple *R* in the population equals zero (0). The model reached statistical significance [*F* = (2, 414) = 151.04, *Sig* = 0.000, this really means *P* < 0.05].

**TABLE 5:** ANOVA for psychological well-being, loving pedagogy, work engagement.

Model		Sum of squares	df	Mean square	F	Sig.
1	Regression	53,704.39	2	26,852.19	151.04	0.000
	Residual	73,601.26	414	177.78		
	Total	127,305.65	416			

*^a^Dependent variable: WE.*

*^b^Predictors: (Constant), PWB and LP.*

To measure whether the independent variables (teachers’ psychological well-being and loving pedagogy) can predict the dependent variable (teachers’ work engagement), the sig. column was studied. As Table 6 reveals, only psychological well-being, as an independent variable, is the significant predictor (*B* = 0.62, *sig* = 0.000). Regarding the other independent variable, loving pedagogy, the results proved that it is not a significant predictor (*sig* = 0.516).

**TABLE 6:** Coefficients for psychological well-being, loving pedagogy, work engagement.

Model		Unstandardized coefficients		Standardized coefficients		
		*B*	Std. error	Beta	*T*	Sig.
1	(Constant)	1.801	5.746		0.313	0.754
	PWB	0.876	0.081	0.621	10.867	0.000
	LP	0.052	0.080	0.037	0.650	0.516

*^a^Dependent variable: WE.*

## Discussion

This study aimed to investigate the relationships between Chinese EFL teachers’ well-being, loving pedagogy, and their work engagement. However, the role of EFL teachers’ psychological well-being and loving pedagogy in predicting their engagement was examined. To do so, three types of questionnaires were distributed among Chinese EFL teachers. The results indicated that there was a significant relationship between teachers’ psychological well-being and engagement in educational contexts. Moreover, the findings showed that teachers’ psychological well-being significantly predicted work engagement. The results also showed that loving pedagogy did not significantly predict teacher engagement. However, there was a significant correlation between these two constructs.

The findings of this study tie well with Greenier et al. (2021), who found out that teachers’ psychological well-being contributes to work engagement. The broad-and-build theory can explicate the reason behind this significant correlation. Teachers’ satisfaction can foster individual competencies, which, in turn, develop psychological well-being and instructional efficiency. They argued that educators’ work engagement and well-being in educational contexts are associated with their job satisfaction. This finding boosts the developing realm of positive psychology and its use in education and instruction, demonstrating how Chinese EFL educators’ psychological well-being, as a feature of positive emotional experience, enhances teachers’ job involvement in academic context. Furthermore, the significant correlation between Chinese EFL educators’ psychological well-being and their work engagement is partially consistent with the results of the study, which confirms the predictability of Sudibjo and Sutarji’s (2020) educators’ psychological well-being to their work engagement. A similar pattern of results was obtained in Shuck and Reio’s (2014) study which highlighted the significance of individuals’ positive perception of constructive work context. Their study indicated that positive emotional workplace ambiance and high involvement tend to enjoy from a widened provision of psychological well-being. Earlier studies verified the positive and significant role of loving pedagogy in learners’ academic, social, and psychological engagement. This study corroborates the significant relationship between loving pedagogy and teachers’ work engagement. However, the predictability power of loving pedagogy was not significant. Several reasons such as cultural differences, methodology, techniques, individual differences, personality traits, etc., can be mentioned for this issue.

This study has some implications for teachers, teacher educators, and educational policy-makers. First, when teachers experience well-being, greater job satisfaction, the positive relationship with learners, and performance feedback at work, they may be more likely to find a way to make their work more pleasant, participate in their workplace decision in order to increase their involvement in their work and workplace, and thus increase their engagement to their work. Therefore, through strong psychological well-being, loving pedagogy, and work engagement relationships, teachers appear better equipped to cope with challenges at work place and show to understand their work more meaningful. Further to the point made above, the significant impact of psychological well-being and pedagogical love on work engagement among Chinese EFL teachers can influence their level of contribution to enhance the performance of their schools and institutes.

Teacher educators can suggest teachers incorporate pedagogical love in their methodology, and realize learners’ requirements, longings, desires, or their motivational factors that encourage them. Moreover, teacher educators can also emphasize instructors attach importance to the constant critical thinking about their relationship with learners in their instructional method. Teacher educators should suggest instructors to be mindful of how they talk to learners, particularly before their peers. Moreover, they can suggest that instructors should listen to students and ask them questions to show you are interested in them. They can do this in class discussions, or through individual or small group meetings.

In order to develop teacher well-being, teacher educators can hold workshops and suggest in-service and pre-service teachers to adopt a growth mindset in their instruction through which they can accept their mistakes and use new learning methods. They can also suggest teachers highlight kindness in their instruction to develop positive emotional states, and positive rapport, and enhance psychological well-being. The teacher educators should be educated concerning positive psychology, and they should instruct well-being strategies, including resilience, mindfulness, and emotional regulation together with professional academic strategies to their instructors to help lessen the inevitable stress and anxiety that are met in their profession.

To improve teachers’ work engagement, teacher educators and mentors can emphasize instructors attach importance to the constant academic development and critical thinking to enhance their instructional method. Instructors should be directed to be well-informed about instructive issues and take advantage of improved learning chances. It is also suggested that teacher educators highlight interaction tools, like mobile applications, which encourage teachers and learners to interact and scaffold that increase efficacy. They should develop confidence and competence among in-service teachers to entice learners’ interests and engage them in the learning process. Teacher educators can enquire about syllabus, education, and schedules to engage them in thinking about educational conditions. They should pay attention to their syllabus designs, and include their opinions in their decision-making about syllabus designs. They can also prove a context for teachers to engage in learner-centered projects.

Also, the educational policy-makers should actively cater occasions for educators to effectively work together with teacher educators, therefore improving communication or network support among them. Social programs, like meetings, can be held to endorse mutual understanding and care or emotional support among teachers. In fact, by knowing about these variables, policymakers would be able to make teachers enthusiastic and improve their level of engagement.

A number of recommendations for future research are given. Studies can be done on teachers from different places, technology-supported contexts, numerous cultural backgrounds, and different educational experiences. Moreover, the in-depth interviews might apply to future studies to triangulate the results. The relationship between pedagogical love and positive psychological constructs such as grit, resilience, and collective efficacy can be suggested for further research. Moreover, the relationship between pedagogical love and teachers’ emotional intelligence can pave the way for positive psychologists. The effect of pedagogical love on teacher burnout can also be another facet that should be scrutinized in the future. Regarding well-being, the impact of teacher well-being on the enhancement of language learners’ skills should be examined in upcoming studies. Further studies are required to specify teachers’ loving pedagogy and well-being in traditional and digital contexts to clear up the effect of contexts on teachers’ positive emotions. Also, the upcoming studies about the effect of gender on pedagogical love and teachers’ psychological well-being can be useful for educational investigators.

## Data Availability Statement

The original contributions presented in this study are included in the article/supplementary material, further inquiries can be directed to the corresponding author.

## Ethics Statement

The studies involving human participants were reviewed and approved by Jilin University Academic Ethics Committee. The patients/participants provided their written informed consent to participate in this study.

## Author Contributions

Both authors listed have made a substantial, direct, and intellectual contribution to the work, and approved it for publication.

## Conflict of Interest

The authors declare that the research was conducted in the absence of any commercial or financial relationships that could be construed as a potential conflict of interest.

## Publisher’s Note

All claims expressed in this article are solely those of the authors and do not necessarily represent those of their affiliated organizations, or those of the publisher, the editors and the reviewers. Any product that may be evaluated in this article, or claim that may be made by its manufacturer, is not guaranteed or endorsed by the publisher.
